# Prevalence of Trachoma in 72 Districts of Afghanistan in 2018−2019: Results of 35 Population-based Prevalence Surveys

**DOI:** 10.1080/09286586.2021.2015784

**Published:** 2022-03-10

**Authors:** Ahmad Shah Salam, Rafiqullah Qayumi, Abdul Majeed Siddiqi, Mohammad Naseem, Mirwais Mansoor, Robert Butcher, Ana Bakhtiari, Kristen Renneker, Rebecca Willis, Cristina Jimenez, Michael Dejene, Naimullah Safi, Anne Heggen, Anthony W. Solomon, Emma M. Harding-Esch, Najeebullah Alizoi

**Affiliations:** a Ministry of Public Health, Kabul, Afghanistan; b HealthNet Tpo, Kabul, Afghanistan; c Clinical Research Department, London School of Hygiene & Tropical Medicine, London, UK; d Task Force for Global Health, Decatur, Georgia, USA; e Sightsavers International, Haywards Heath, UK; f Sightsavers International, Addis Ababa, Ethiopia; g Afghanistan Country Office, World Health Organization, Kabul, Afghanistan; h Fred Hollows Foundation; i Department of Control of Neglected Tropical Diseases, World Health Organization, Geneva, Switzerland

**Keywords:** Trachoma; Afghanistan; blindness; neglected tropical diseases; prevalence

## Abstract

**Background:**

To determine where interventions are needed to eliminate trachoma as a public health problem, prevalence data are needed. We aimed to generate baseline population-based data on trachoma prevalence in suspected-endemic areas of Afghanistan.

**Methods:**

Cross-sectional population-based prevalence surveys designed according to World Health Organization (WHO) recommendations were conducted in 35 evaluation units (EUs) covering 72 districts. In selected households, all resident individuals aged ≥1 year were examined for trachomatous inflammation—follicular (TF) and trachomatous trichiasis (TT) according to the WHO simplified trachoma grading system. Water, sanitation and hygiene access was assessed in households of survey participants.

**Results:**

104,104 people aged ≥1 year were examined, including 43,774 children aged 1–9 years and 46,439 people aged ≥15 years. The age-adjusted prevalence of TF in 1–9-year-olds was ≥5% in 3 EUs, with the highest EU TF prevalence being 7.8%. The age- and gender-adjusted prevalence of TT unknown to the health system in ≥15-year-olds was <0.2% in all EUs. The majority of households had access to an improved water source within 30 minutes of the house. However, only a minority of households had an improved latrine and/or a handwash station.

**Conclusions:**

Trachoma is not a public health problem in the majority of EUs surveyed. However, antibiotic mass drug administration, promotion of facial cleanliness and environmental improvement (the A, F and E components of the SAFE strategy) are needed for trachoma elimination purposes in three of the EUs surveyed in Afghanistan.

## Introduction

Trachoma is a neglected tropical disease (NTD) and the world’s leading infectious cause of blindness.^1^ It is caused by infection of the eye with *Chlamydia trachomatis*, which causes inflammation of the inner eyelid. Ocular *C. trachomatis* is thought to be spread from person to person through contact with contaminated hands, clothing and bedding, and by eye-seeking flies.^2–4^ Because of these modes of transmission, trachoma disproportionately affects those who live in crowded conditions, those who lack ready access to water to regularly clean their faces and those with inadequate sanitation facilities, with open defaecation encouraging fly breeding.^5^ Ocular *C. trachomatis* is often first contracted in early childhood. It is associated with a conjunctivitis known as active trachoma, which includes the signs trachomatous inflammation—follicular (TF) and/or trachomatous inflammation—intense (TI). Trachoma’s pathogenetic cascade takes many years and multiple rounds of infection and inflammation to become clinically important.^6^ In severe cases, the eyelids turn inwards so that the eyelashes scratch the surface of the eyeball (trachomatous trichiasis, TT), causing pain and, in some cases, irreversible blindness. Poor eyesight from trachoma has a direct impact on livelihoods by limiting contributions to the economic output of a household and through indirect reductions in productivity of other household members who act as carers.^7^

Trachoma is considered a public health problem when the district-level prevalence of TT unknown to the health system in people aged ≥15 years is ≥0.2% and/or the prevalence of TF in 1–9-year olds is ≥5%.^8^ Trachoma can be treated and ultimately eliminated as a public health problem by implementing the World Health Organization (WHO)-endorsed SAFE strategy (surgery, antibiotics, facial cleanliness and environmental improvement).^9^ The WHO Alliance for the Global Elimination of Trachoma by 2020 (GET2020) set a goal of eliminating trachoma as a public health problem in all endemic countries by 2020, using the SAFE strategy.^8,10,11^ The target date was revised to 2030 in the recently-published NTD road map 2021 − 2030.^12^

In the Eastern Mediterranean region as a whole, the principal causes of vision loss are refraction and accommodation disorders, cataract, glaucoma, and macular degeneration.^13^ Afghanistan has a majority rural population (75% according to the Afghanistan Demographic and Health Survey 2015^14^) many of whom live in conditions amenable to the transmission of trachoma. According to the Afghanistan Demographic and Health Survey 2015, 86% percent of urban households have access to an improved drinking water source, in contrast to only 58% of rural households, while only 25% have access to improved sanitation facilities, suggesting there may be a large population at risk of trachoma.^14,15^

In 2018, 109 of the 421 districts in Afghanistan were either suspected to be trachoma endemic or neighbouring suspected trachoma-endemic districts. Of these, 32 were considered too insecure for survey during the phase of activities presented in this work. Of the remaining 77, 43 were suspected to be trachoma endemic because cases of TF and/or TT had been identified through established health programmes. These 43 suspected-endemic districts comprised three districts identified through a trachoma control program in 2006–2008, 20 districts identified through outreach activities in 2011–2013, and 20 districts identified through hospital records in 2014. In Afghanistan, people often come from multiple districts to attend specialised hospital or outreach services and the location of residence of patients with trachoma was not recorded. Therefore, while evidence of trachoma was found in 43 districts, it is unlikely that cases of trachoma are limited to these particular districts. For that reason, an additional 34 districts neighbouring the 43 suspected-endemic districts were also felt to be in need of trachoma mapping. As such, a total of 77 districts were targeted for baseline prevalence surveys during this first phase of activities. These 77 districts were spread across 16 provinces of Afghanistan with a combined population of over 6.4 million.

The aim of this study was to estimate the prevalence of trachoma in suspected-endemic districts in Afghanistan using protocols consistent with WHO recommendations. The data will guide what steps, if any, are needed to eliminate trachoma as a public health problem in Afghanistan.

## Methods

This report conforms with the Strengthening Reporting of Observational Studies in Epidemiology guidelines.^16^

### Study ethics

Surveys adhered to the tenets of the Declaration of Helsinki. The Ministry of Public Health of Afghanistan approved the survey protocol before commencement. Ethical approval for Tropical Data to provide support for these surveys was granted by the London School of Hygiene & Tropical Medicine (16105). Communities were notified in advance of a team’s arrival to ensure they did not arrive unexpectedly. Before being invited to take part, individuals were informed about the nature of the surveys and requirements for taking part. Survey participants provided verbal consent. A parent or guardian provided consent on behalf of individuals under the age of 18 years. Consent for examination was recorded in the Tropical Data app.

### Sample size

In accordance with WHO recommendations,^17,18^ surveys were designed to estimate an expected TF prevalence of 10% in children aged 1–9 years with a precision of ±3% at the 95% confidence level, in each evaluation unit (EU). To achieve this precision, the protocol was designed to facilitate sampling of 1222 children per EU based on a design effect of 2.65 and an inflation factor of 1.2 to account for non-response.^18^

### Participant selection

WHO recommends that trachoma surveys be implemented in EUs, defined as administrative units for healthcare management, usually with populations of 100,000–250,000 people.^19^ Health services in Afghanistan are usually delivered at district level. However, due to the small size of some districts, in several cases in this survey series, we grouped multiple districts to be surveyed as a single EU (Supplementary Table S1). In these instances, we ensured that the districts were geographically, socio-economically and culturally similar. Of the 77 districts described in the introduction, none had previously had formal population-based prevalence surveys or recent trachoma elimination interventions. Five districts in need of mapping were not surveyed in this exercise. First, two districts (Hazarsumuch and Hazrat-e-Sultan) could not be combined with other districts to form a geographically contiguous EU with a population of 100,000–250,000. Second, three districts (Baharak and Namakab in Takhar province and Tagab in Kapisa province) were considered too insecure to include in this series. The 72 remaining districts were grouped into 35 EUs.

Individuals were selected for recruitment using two-stage cluster sampling. The primary sampling unit was the village (hereafter referred to as the cluster), selected systematically with a probability-proportional-to-size method. The secondary sampling unit was the household, selected using compact segment sampling in each selected cluster. All individuals aged ≥1 year in each selected household were eligible to take part in the survey.

The number of clusters to survey per EU was determined by dividing the number of children to sample in the EU as a whole by the product of the expected number of 1–9-year-olds per household and the number of households to sample per cluster. For logistical reasons, the number of households to sample per cluster was determined to be the number a team could feasibly survey in one day; in Afghanistan, this was 25 households. Recent demographic surveys suggested there would be approximately eight people per household, and approximately 28% of the population would be 1–9 years old,^14^ therefore we expected a mean of 2.2 children aged 1–9 years per household. Based on this, 22 clusters were selected per EU.

### Clinical examination

Survey teams had successfully completed the internationally-standardised Tropical Data training scheme.^20^ Briefly, this involves graders taking part in a classroom-based introduction to the diagnosis of trachoma according to the WHO simplified grading system,^21^ achieving an inter-grader agreement (IGA) score of ≥0.7 for the diagnosis of TF against expert consensus on a set of 50 photographs, and achieving an IGA score for TF of ≥0.7 against a Tropical Data-certified grader trainer in the assessment of 50 eyes of children, in the field.^18^ Data recorders were trained in accurate data collection of clinical signs and household water, sanitation and hygiene (WASH) variables using the Secure Data Kit-based Tropical Data app.

All consenting individuals aged one year or above in selected households were examined for the clinical signs TF, TI and TT. Follicle size guides were used to help maintain quality of diagnosis of TF.^22^ Participants with TT were also graded for trachomatous scarring (TS) and asked whether they had been offered management, in the form of surgery or epilation. Participants with TT who either reported never having been offered management in at least one eye with TT or could not remember whether they had been offered management for TT were classed as having TT ‘unknown to the health system’. Graders received at least one supervisory visit in the field by an experienced trachoma grader who had been certified by Tropical Data as a grader trainer over the course of the surveys to ensure they were maintaining a high standard of clinical grading.

### Water, sanitation and hygiene access

In each household, the data recorder worked with the household head to complete a questionnaire on the type and proximity of sources of water used for washing and drinking, the type of latrine the household used and whether it was shared with neighbouring households, and whether there was a handwash station with soap and water within 15 m of the household latrine. (The latter question was not asked if the household did not have a latrine.) The questionnaire was a version of the WHO/United Nations Childrens' Fund (UNICEF) Joint Monitoring Programme (JMP) for Water Supply and Sanitation WASH household questionnaire adapted for trachoma surveys.^18^ Where reasonably accessible, water sources, sanitation facilities and handwash stations were assessed by direct observation.

### Data analysis

Data were collected, managed and analysed with the support of Tropical Data (www.tropicaldata.org), which ensured the surveys were subject to the same stringent quality control and quality assurance protocols as surveys in other countries.^23^ Prevalence estimates were generated using the Global Trachoma Mapping Project methodology.^18^ Age-specific TF proportions were adjusted using age weights from the most recent census, in one-year age bands, at the cluster level. Age-specific TT proportions were adjusted using age and gender weights from the most recent census, in five-year age bands, at the cluster level. EU-level disease prevalence was calculated as the mean adjusted cluster-level proportions of TF and TT, respectively. The 95% confidence interval (CI) was determined using the 97.5th and 2.5th centiles of the distribution of 10,000 resamples (with replacement) of the adjusted cluster-level proportions. For data analysis of WASH variables, categories were created in accordance with WHO/UNICEF JMP definitions of improved and unimproved facilities.^24^

Association between TF and individual- and household-level variables was examined in children aged 1–9 years using a multi-level mixed-effects multivariable logistic regression model (lme4::glmer function in R,^25^) as used elsewhere.^26^ Null models with EU, cluster and household-level random effects were compared using likelihood ratio tests (LRTs) to determine which clustering level should be included as random effects. Multivariable models were composed of variables with a significant relationship with TF (*p* < .0.5 for LRT between null model and model containing single fixed effect variable) in univariate analysis and variables which significantly improved model fit (measured by comparison of log-likelihood, akaike information criterion and LRT between models with and without each fixed-effect independent variable) on stepwise addition of variables.

Due to the small number of cases, association between TT and potential risk factors was assessed in people aged 15 years and over using a binomial generalised linear model without a random-effects variable being included. Age and gender were included as independent variables in that model but household-level WASH variables were not, because the WASH environment captured during this study does not necessarily reflect the lifetime accumulated risk.

## Results

### Study population

A total of 35 EUs encompassing 72 districts across 16 provinces were surveyed in two phases between September 2018–December 2019. A total of 106,202 people aged ≥1 year were enumerated, of whom 104,104 (98%) were examined. Of 2,098 non-participants, 1,021 (49%) were absent, 866 (41%) refused and the remaining 211 (10%) recorded another unspecified reason for not taking part. The province-level participation rate is shown in Table 1.

**Table 1.** Population aged ≥1 year enrolled in trachoma prevalence surveys in Afghanistan, September 2018–December 2019.

Province	Number of EUs	Survey population (aged ≥1 year)					
		Enumerated	Absent	Refused	Other	Examined	% female examined
Baghlan	3	8,970	48	87	0	8,835	47
Bamyan	2	6,095	22	36	0	6,037	52
Day Kundi	2	5,911	34	44	0	5,833	46
Faryab	2	6,566	57	35	0	6,474	47
Hirat	2	5,904	129	46	0	5,729	46
Kapisa	2	5,855	41	88	0	5,726	45
Kunar	2	6,993	79	1	1	6,912	42
Kunduz	4	11,969	65	25	0	11,879	39
Laghman	2	6,349	43	9	3	6,294	43
Logar	2	6,298	40	7	6	6,245	36
Nangarhar	1	3,530	54	257	198	3,021	44
Nimroz	1	2,821	59	8	0	2,754	51
Paktya	2	5,198	74	4	0	5,120	37
Samangan	2	6,005	67	62	0	5,876	48
Sari Pul	2	5,140	15	0	0	5,125	46
Takhar	4	12,598	194	157	3	12,244	49
*Total*	*35*	*106,202*	*1,021*	*866*	*211*	*104,104*	*45*

EU: evaluation unit.

Of those enumerated, 44,227 (42%) were aged 1–9 years. A total of 43,774 (99%) of these were examined. Of the 453 child non-respondents, 283 (62%) refused to take part. 47,964 enumerated individuals were aged ≥15 years. Of those, 46,439 were examined (97%). 756 (50%) of 1,525 non-respondent ≥15-year-olds were absent at the time the teams visited. At the EU level, the participation rate ranged from 96–100% and 69–100% in participants aged 1–9 years and ≥15 years, respectively. The gender balance in survey respondents was overall better in children than in adults. 48% (range: 42–53%) of 1–9-year-old participants were female whereas 41% (range: 13–64%) of ≥15-year-old participants were female. The EU-level participation rate broken down by age group is shown in Supplementary Table S1.

### Clinical data

**Figure 1.**
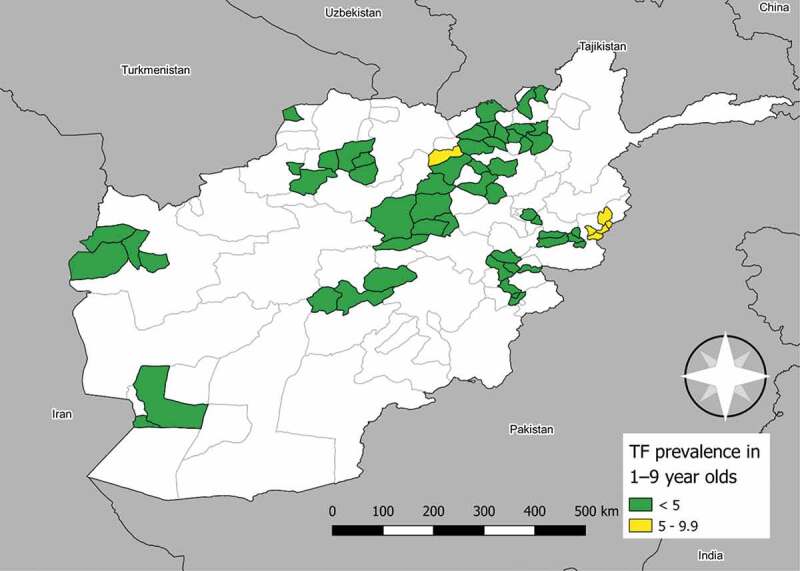
Prevalence of trachomatous inflammation—follicular (TF) in children aged 1–9 years in Afghanistan. Surveys conducted from September 2018–December 2019. The boundaries and names shown and the designations used on this map do not imply the expression of any opinion whatsoever on the part of the authors, or the institutions with which they are affiliated, concerning the legal status of any country, territory, city or area or of its authorities, or concerning the delimitation of its frontiers or boundaries.

Overall, the prevalence of TF was low. In 43,774 children aged 1–9 years, 689 cases of TF and 122 cases of TI were identified during this study. The age-adjusted prevalence of TF in children aged 1–9 years was ≥5% in 3 of the 35 surveyed EUs. These EUs encompassed the following districts: Asad Abad and Marawara (EU-level prevalence of TF: 5.2%, 95% confidence interval [CI]: 3.2 − 7.7), and Chawkay, Khas Kunar and Sirkanay (7.8%, 95% CI: 6.1 − 9.6) in Kunar Province; and Aybak, Dara-I-Sufi Payan and Feroznakhchir districts (5.9%, 95% CI: 3.2 − 8.7) in Samangan Province. The median age-adjusted prevalence of TF in children aged 1–9 years was 0.7% (range: 0–4.7%) in the remaining 32 districts. The median age-adjusted EU-level prevalence of TI in all 35 EUs was 0.1% (range: 0.0–1.4%). TF and TI prevalence were well correlated (Pearson’s correlation coefficient: 0.86). The age-adjusted prevalence of TF is shown in Figure 1, and the age-adjusted prevalence of TF and TI, including numerators and denominators per EU, is shown in Table 2.

**Table 2.** Age-adjusted trachomatous inflammation—follicular (TF) and trachomatous inflammation—intense (TI) prevalence in children aged 1–9 years in Afghanistan, September 2018–December 2019.

District(s)	Number examined (1–9 years)	Number of TF cases	Age-adjusted TF prevalence (%)	TF prevalence 95% confidence interval	Number of TI cases	Age-adjusted TI prevalence (%)	TI prevalence 95% confidence interval
Baghlani Jadid	1,519	2	0.3	0.0–0.8	1	0.3	0.0–0.8
Nahrin, Puli Khumri	1,090	6	0.4	0.2–0.8	7	0.5	0.2–0.9
Bamyan, Kahmard, Sayghan, Shibar	962	12	1.3	0.1–2.5	1	0.1	0.0–0.2
Gizab, Kijran, Kiti, Nili	1,209	30	1.8	0.8–2.7	6	0.4	0.1–0.8
Bilchiragh, Maymana	1,031	51	4.7	2.6–6.8	8	1.0	0.3–1.9
Ghoryan, Kohsan	987	9	0.7	0.1–1.4	0	0.0	-
Hesa Awal Kohistan, Hesa Duwum Kohistan	961	8	0.7	0.3–1.2	6	0.5	0.1–1.0
Asad Abad, Marawara	1,552	81	5.2	3.2–7.7	12	0.7	0.3–1.2
Imam Sahib	1,284	21	1.3	0.8–2.0	1	0.1	0.0–0.3
Kunduz	1,290	4	0.2	0.0–0.5	0	0.0	-
Mihtarlam	1,219	18	1.4	0.5–2.3	0	0.0	-
Qarghayi	1,880	38	2.5	1.6–3.4	8	0.3	0.1–0.6
Puli Alam	1,762	52	2.6	1.7–3.5	9	0.4	0.1–0.8
Dara-I-Nur, Kama, Kuz Kunar	1,970	14	0.8	0.3–1.4	9	0.5	0.2–0.9
Chakhansur, Zaranj	1,219	3	0.4	0.0–0.8	0	0.0	-
Ahmadaba, Jaji, Lija Ahmad Khel	1,457	2	0.1	0.0–0.3	1	0.1	0.0–0.2
Gardez, Jadran, Shwak	1,384	7	0.4	0.0–0.7	0	0.0	-
Aybak, Dara-I-Sufi Payan, Feroznakhchir	965	52	5.9	3.2–8.7	10	1.4	0.2–3.5
Sangcharak, Sozma Qala	1,320	4	0.7	0.0–1.8	0	0.0	-
Sari Pul, Sayyad	1,353	15	1.1	0.3–1.9	3	0.2	0.0–0.4
Taluqan	963	5	1.0	0.2–2.2	0	0.0	-
Dushi, Khinjan	1,263	0	0.0	-	3	0.2	0.0–0.3
Panjab, Yakawlang	1,000	0	0.0	-	0	0.0	-
Miramor, Shahristan	1,234	0	0.0	-	0	0.0	-
Andkhoy, Qorghan	827	6	0.5	0.2–0.9	1	0.1	0.0–0.3
Guzara, Zinda Jan	1,147	6	0.4	0.1–0.8	1	0.1	0.0–0.3
Alasay, Nijrab	1,409	0	0.0	-	0	0.0	-
Chawkay, Khas Kunar, Sirkanay	1,718	134	7.8	6.1–9.6	23	1.2	0.6–1.7
Ali Abad, Chahar Dara	1,216	16	1.2	0.5–1.7	0	0.0	-
Khan Abad	1,403	0	0.0	-	0	0.0	-
Khushi, Muhammad Agha	1,348	18	1.2	0.7–2.0	1	0.1	0.0–0.2
Dara-I-Sufi Balla, Khuram Wa Sarbagh, Ruyi Du Ab	954	36	2.7	1.6–4.2	7	0.6	0.2–1.1
Bangi, Chal	892	5	0.5	0.1–1.0	1	0.1	0.0–0.1
Chah Ab, Yangi Qala	998	9	0.7	0.2–1.4	0	0.0	-
Farkhar, Kalafgan	988	25	2.6	1.5–4.0	3	0.2	0.0–0.5
*Total*	*43,774*	*689*	-	-	*122*	-	-

Overall, the prevalence of TT was low. Of 46,439 individuals aged ≥15 years examined, 24 cases of TT were identified, 18/24 (75%) of which were in women. Fourteen (58%) of 24 individuals with TT had not been offered management or could not remember being offered management, so were classified as having TT unknown to the health system. Just under half (11/24) of the TT cases had TS recorded. The age- and gender- adjusted prevalence of TT unknown to the health system in people aged ≥15 years was <0.2% in all 35 surveyed EUs. The adjusted prevalence of TT unknown to the health system in people aged ≥15 years was >0% in 8 EUs, and the maximum adjusted TT unknown to the health system prevalence in people aged ≥15 years was 0.16% (95% confidence interval: 0.00–0.40%), found in the EU comprising Guzara and Zinda Jan districts of Hirat province (Table 3).

**Table 3.** Age- and gender-adjusted prevalence of trachomatous trichiasis (TT) unknown to the health system among people aged ≥15 years in Afghanistan, September 2018–December 2019.

District(s) in EU	Number of examined (≥15 years)	Number of TT cases	Number of TT cases with TS	Number of TT cases unknown to the health system	Adjusted prevalence of TT unknown to the health system	95% confidence interval
Baghlani Jadid	1,056	0	0	0	0.00	-
Nahrin, Puli Khumri	1,177	0	0	0	0.00	-
Bamyan, Kahmard, Sayghan, Shibar	1,232	0	0	0	0.00	-
Gizab, Kijran, Kiti, Nili	1,006	0	0	0	0.00	-
Bilchiragh, Maymana	1,606	2	1	0	0.00	-
Ghoryan, Kohsan	1,376	2	2	2	0.08	0.00–0.17
Hesa Awal Kohistan, Hesa Duwum Kohistan	897	1	1	0	0.00	-
Asad Abad, Marawara	1,191	2	0	2	0.08	0.00–0.25
Imam Sahib	949	0	0	0	0.00	-
Kunduz	1,184	0	0	0	0.00	-
Mihtarlam	1,049	9	2	3	0.08	0.00–0.19
Qarghayi	1,745	0	0	0	0.00	-
Puli Alam	1,183	0	0	0	0.00	-
Dara-I-Nur, Kama, Kuz Kunar	1,047	0	0	0	0.00	-
Chakhansur, Zaranj	1,144	1	1	1	0.08	0.00–0.23
Ahmadaba, Jaji, Lija Ahmad Khel	673	0	0	0	0.00	-
Gardez, Jadran, Shwak	748	0	0	0	0.00	-
Aybak, Dara-I-Sufi Payan, Feroznakhchir	1,341	0	0	0	0.00	-
Sangcharak, Sozma Qala	1,058	0	0	0	0.00	-
Sari Pul, Sayyad	926	1	0	1	0.12	0.00–0.36
Taluqan	1,286	2	0	2	0.07	0.00–0.19
Dushi, Khinjan	1,528	0	0	0	0.00	-
Panjab, Yakawlang	1,970	0	0	0	0.00	-
Miramor, Shahristan	1,477	0	0	0	0.00	-
Andkhoy, Qorghan	2,012	0	0	0	0.00	-
Guzara, Zinda Jan	1,316	2	2	2	0.16	0.00–0.40
Alasay, Nijrab	1,583	0	0	0	0.00	-
Chawkay, Khas Kunar, Sirkanay	1,461	1	1	1	0.08	0.00–0.25
Ali Abad, Chahar Dara	1,531	1	1	0	0.00	-
Khan Abad	1,221	0	0	0	0.00	-
Khushi, Muhammad Agha	1,367	0	0	0	0.00	-
Dara-I-Sufi Balla, Khuram Wa Sarbagh, Ruyi Du Ab	1,827	0	0	0	0.00	-
Bangi, Chal	1,734	0	0	0	0.00	-
Chah Ab, Yangi Qala	1,755	0	0	0	0.00	-
Farkhar, Kalafgan	1,783	0	0	0	0.00	-
*Total*	*46,439*	*24*	*11*	*14*	-	-

EU: evaluation unit; TS: trachomatous scarring.

### Access to water, sanitation and hygiene

**Figure 2.**
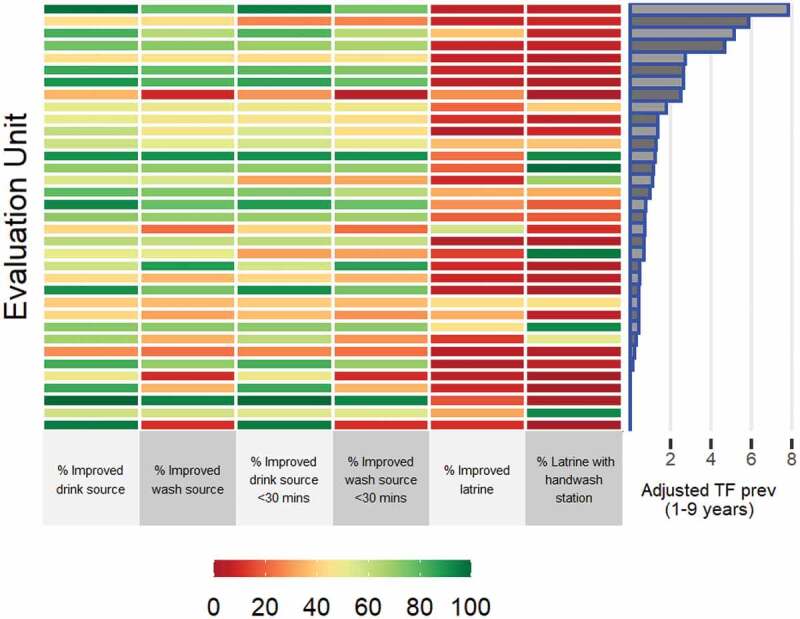
Percentage of households with improved water, sanitation and hygiene facilities in evaluation units surveyed for trachoma in Afghanistan in September 2018–December 2019. Data are presented in descending order of evaluation unit-level age-adjusted trachomatous inflammation—follicular (TF) prevalence (%) in children aged 1–9 years from top to bottom.

A total of 770 clusters and 18,864 households (22 clusters per EU, a median of 549 households per EU) were visited across the 35 EUs. Water access was generally good. The median proportion of households per EU with an improved drinking water source was 62% (range: 28–100%) and the median proportion of households per EU with a drinking water source within 30 minutes of the household was 94% (range: 35–100%). Conversely, latrine and handwash station status was poor. The median proportion of households per EU with an improved latrine was 11% (range: 3–57%) and a latrine with a handwash station was 7% (range: 0–99%). Figure 2 illustrates the WASH coverage per EU in the context of TF prevalence in children aged 1–9 years. There was no evidence of a correlation between EU-level TF prevalence and EU-level proportion of households with improved WASH facilities (Pearson’s correlation coefficient range: −0.20–0.19) (Supplementary Table S2).

### Individual and household variables associated with TF and TT

There were 247 children living in households where either the water source or latrine could not be categorised by the recorders in the field. For the purposes of the regression analyses, these data were considered to be missing and these individuals were excluded from the TF association analysis. The analysis was therefore conducted on 43,527 children. EU, cluster and household were all included as random effects parameters following stepwise model assembly (Table 4). TF was more common in older than younger children; compared to the reference 1–3-year-old age group, the adjusted odds ratio (aOR) for finding TF in 4–6-year olds was 5.25 (95% CI: 3.86 − 7.14) and in 7–9-year olds was 7.59 (95% CI: 5.59 − 10.29). TF was also more common in females than males (aOR: 1.62, 95% CI: 1.36–1.92). There was no good evidence of an association between household-level variables and TF.

**Table 4.** Univariable and multivariable multi-level hierarchical binomial mixed-effects regression analyses of variables associated with trachomatous inflammation—follicular (TF) in children aged 1–9 years in Afghanistan, September 2018–December 2019. All models include evaluation unit, cluster and household as random effects parameters.

Variable	Group	No TF	TF	Univariable			Multivariable		
				OR	95% CI	P	aOR	95% CI	P
Age group	1–3 years	14,010	55	Reference		<0.001	Reference		<0.001
	4–6 years	15,103	278	5.25	3.87 − 7.14		5.25	3.86 − 7.14	
	7–9 years	13,730	351	7.58	5.59 − 10.27		7.59	5.59 − 10.29	
Gender	Male	22,343	289	Reference			Reference		<0.001
	Female	20,384	394	1.56	1.32 − 1.84		1.62	1.36 − 1.92	
Number of children aged under 10 years in household	1–2	11,708	156	Reference		0.290	Not tested		
	3–4	22,843	378	1.06	0.85 − 1.32				
	≥5	8,176	149	1.25	0.94 − 1.66				
Washing water source	Improved water source within 30 minutes of household	21,771	381	Reference		0.619	Not tested		
	Improved water source >30 minutes from household	1,429	31	1.12	0.66 − 1.92				
	Unimproved water source	6,567	88	1.21	0.88 − 1.68				
	Surface water	13,076	184	0.97	0.74 − 1.28				
Latrine status	Private, improved latrine	6,839	89	Reference		0.875	Not tested		
	Shared, improved latrine	610	11	1.33	0.62 − 2.87				
	Unimproved latrine	27,281	434	1.03	0.76 − 1.39				
	Open defecation	8,113	150	0.97	0.66 − 1.43				

* 247 children, including five children with TF, were living in households where the water and/or sanitation facilites could not be classified and were omitted from this analysis.

aOR: adjusted odds ratio; CI: confidence interval; OR: odds ratio; TF: trachomatous inflammation—follicular.

All examined participants had age and gender data available for TT regression analyses. Cluster was the only random effects variable included in the TT regression models. TT was much more common in the ≥65-year-olds than the under 65-year-olds (aOR: 35.40; 95% CI: 12.57 − 99.65; likelihood ratio test [LRT]: *p* < .001) and females (aOR: 6.17; 95% CI: 2.25 − 16.91; LRT: *p* < .001).

## Discussion

We aimed to assess EU-level prevalence of trachoma in districts that had not previously received trachoma elimination activities but where cases had previously been found in routine healthcare work. We demonstrated that in 32/35 EUs surveyed, trachoma is not a public health problem according to WHO criteria. According to those criteria, interventions to reduce active trachoma prevalence and ocular *C. trachomatis* transmission (that is, the A, F and E components of the SAFE strategy) are not needed in the districts comprising these 32 EUs. The prevalence of TF was ≥5% (but <10%) in three EUs, which covered eight districts and contained a total estimated population of 421,760. A single round of antibiotic mass drug administration (MDA) is warranted in these EUs to reduce the prevalence of TF to below 5%.^27^ Efforts should also be made to address facial cleanliness and environmental improvement in these EUs, to reduce *C. trachomatis* transmission and ensure infection does not re-emerge post-MDA. At least six months after the single round of MDA, a trachoma impact survey should be conducted to determine whether the interventions have reduced the prevalence of TF to below the elimination target.^28^

Despite our encouraging results, a number of challenges still face the Afghanistan National Trachoma Task Force. First, there are a number of districts that were not surveyed in this series but where trachoma is suspected of being endemic. This includes five of the 77 districts which were identified for this series but were either not able to be combined into EUs or could not be surveyed due to insecurity: mapping activities are still required in these five districts. In addition to the 72 surveyed and five outstanding districts from this series, a further 32 districts in three provinces, with an estimated combined population of 2.5 million people, are in need of mapping, but were too insecure for survey teams at the time of this first phase of activities. Second, although the prevalence of TT unknown to the health system was <0.2% in all EUs studied, implementation of a strategy to identify and manage incident cases of TT will be needed in previously endemic districts to gain validation of elimination of trachoma as a public health problem. Finally, while most EUs studied here reported a low prevalence of trachoma, the vast majority of households were found to have poor access to sanitation and handwashing facilities. The relationship between trachoma and WASH facilities at the individual level in these surveys was weak. This does not imply there is no relationship between WASH facilities and trachoma: the relationship between facility availability, use of facilities and transmission of infection is complex and would not be fully captured by the facility assessment carried out during these surveys. This also does not negate the need for improvements in WASH provision, as planning and implementation of the F and E elements of the SAFE strategy are conducted based on EU-level prevalence, rather than individual-level association. However, given the low prevalence of TF in many EUs, addressing the limited WASH facility coverage may be beyond the direct scope of the trachoma programme. These data could help local authorities to plan and advocate for improvements to WASH facility coverage for Afghanistan’s rural population as part of national efforts to move towards achieving United Nations Sustainable Development Goal 6. The cross-sectoral benefits of such improvements are reflected in the NTD road map 2021−2030, and tools such as the NTD Non-governmental organisation Network (NNN)-WHO WASH toolkit are available to provide guidance for programmes on relevant activities.^12,29^

There were limitations to this study. First, the definition of TT has been extensively discussed at international meetings of policymakers for trachoma. During the fourth Global Scientific Meeting on Trachoma held in November 2018, it was agreed that misdirected lashes must originate from the upper lid for a trichiasis case to be defined at TT.^30,31^ The data collection tool used in this survey did not differentiate upper from lower lid trichiasis, therefore we cannot determine what proportion of the identified TT cases had upper lid involvement. Second, as indicated above, there were suspected endemic populations that could not be covered in this exercise due to security concerns, therefore the national picture of trachoma endemicity is not yet complete in Afghanistan.

## Conclusions

These data are the first published record of population-based trachoma prevalence surveys in Afghanistan. This represents a major step forward for the Afghanistan trachoma elimination programme, and will hopefully give momentum to the wider NTD elimination agenda in the country. By identifying EUs in which to focus elimination efforts, Afghanistan can apply for an azithromycin donation from Pfizer through the International Trachoma Initiative, advocate for partner support to improve WASH infrastructure, and begin to plan for national elimination as a public health problem.

## Supplementary Material

Supplemental Material
