# Supplementary material for: Prevalence of Trachoma in 72 Districts of Afghanistan in 2018−2019: Results of 35 Population-based Prevalence Surveys
Source: Ophthalmic Epidemiol. 2022 Mar 10;30(6):608–18. doi: 10.1080/09286586.2021.2015784 (PMC10581673; doi:10.1080/09286586.2021.2015784)
Supplement: Supplemental Material [file IOPE_A_2015784_SM1015.zip › Afghanistan_trachoma_revised_supplementary_material.docx]

**Prevalence of trachoma in 72 districts of Afghanistan in 2018−2019: results of 35 population-based prevalence surveys.**

Ahmad Shah Salam, Rafiqullah Qayumi, Abdul Majeed Siddiqi, Mohammad Naseem, Mirwais Mansoor, Robert Butcher, Ana Bakhtiari, Kristen Renneker, Rebecca Willis, Cristina Jimenez, Michael Dejene, Naimullah Safi, Anne Heggen, Anthony W. Solomon, Emma M. Harding-Esch, Najeebullah Alizoi.

**Supplementary material**

**Supplementary Table 1.** Population examined during baseline trachoma prevalence surveys in 35 evaluation units (EUs) in Afghanistan, September 2018–December 2019.

| **Province** | **District(s) in EU** | **EU ID** | **Age group (years)** | **Enumerated** | **Absent** | **Refused** | **Other** | **Examined** | **% female examined** |
| --- | --- | --- | --- | --- | --- | --- | --- | --- | --- |
| Baghlan | Baghlani Jadid | 10644 | 1–9 | 1525 | 6 | 0 | 0 | 1519 | 46 |
|  |  |  | ≥15 | 1069 | 11 | 2 | 0 | 1056 | 29 |
|  |  |  | Total | 3013 | 19 | 2 | 0 | 2992 | 39 |
|  | Nahrin, Puli Khumri | 10645 | 1–9 | 1128 | 1 | 37 | 0 | 1090 | 53 |
|  |  |  | ≥15 | 1222 | 0 | 45 | 0 | 1177 | 64 |
|  |  |  | Total | 2754 | 1 | 85 | 0 | 2668 | 57 |
|  | Dushi, Khinjan | 10665 | 1–9 | 1263 | 0 | 0 | 0 | 1263 | 48 |
|  |  |  | ≥15 | 1551 | 23 | 0 | 0 | 1528 | 41 |
|  |  |  | Total | 3203 | 28 | 0 | 0 | 3175 | 44 |
| Bamyan | Bamyan, Kahmard, Sayghan, Shibar | 10646 | 1–9 | 980 | 2 | 16 | 0 | 962 | 53 |
|  |  |  | ≥15 | 1254 | 5 | 17 | 0 | 1232 | 55 |
|  |  |  | Total | 2619 | 7 | 36 | 0 | 2576 | 54 |
|  | Panjab, Yakawlang | 10666 | 1–9 | 1010 | 10 | 0 | 0 | 1000 | 53 |
|  |  |  | ≥15 | 1973 | 3 | 0 | 0 | 1970 | 51 |
|  |  |  | Total | 3476 | 15 | 0 | 0 | 3461 | 51 |
| Day Kundi | Gizab, Kijran, Kiti, Nili | 10647 | 1–9 | 1237 | 7 | 21 | 0 | 1209 | 49 |
|  |  |  | ≥15 | 1025 | 0 | 19 | 0 | 1006 | 50 |
|  |  |  | Total | 2666 | 8 | 44 | 0 | 2614 | 48 |
|  | Miramor, Shahristan | 10667 | 1–9 | 1237 | 3 | 0 | 0 | 1234 | 46 |
|  |  |  | ≥15 | 1496 | 19 | 0 | 0 | 1477 | 41 |
|  |  |  | Total | 3245 | 26 | 0 | 0 | 3219 | 44 |
| Faryab | Bilchiragh, Maymana | 10648 | 1–9 | 1060 | 3 | 26 | 0 | 1031 | 48 |
|  |  |  | ≥15 | 1643 | 35 | 2 | 0 | 1606 | 48 |
|  |  |  | Total | 3241 | 39 | 28 | 0 | 3174 | 49 |
|  | Andkhoy, Qorghan | 10668 | 1–9 | 831 | 0 | 4 | 0 | 827 | 47 |
|  |  |  | ≥15 | 2028 | 14 | 2 | 0 | 2012 | 45 |
|  |  |  | Total | 3325 | 18 | 7 | 0 | 3300 | 45 |
| Hirat | Ghoryan, Kohsan | 10649 | 1–9 | 1026 | 13 | 26 | 0 | 987 | 51 |
|  |  |  | ≥15 | 1469 | 73 | 20 | 0 | 1376 | 51 |
|  |  |  | Total | 2996 | 114 | 46 | 0 | 2836 | 51 |
|  | Guzara, Zinda Jan | 10669 | 1–9 | 1162 | 15 | 0 | 0 | 1147 | 48 |
|  |  |  | ≥15 | 1316 | 0 | 0 | 0 | 1316 | 32 |
|  |  |  | Total | 2908 | 15 | 0 | 0 | 2893 | 41 |
| Kapisa | Hesa Awal Kohistan, Hesa Duwum Kohistan | 10650 | 1–9 | 983 | 3 | 19 | 0 | 961 | 50 |
|  |  |  | ≥15 | 965 | 0 | 68 | 0 | 897 | 48 |
|  |  |  | Total | 2308 | 4 | 88 | 0 | 2216 | 50 |
|  | Alasay, Nijrab | 10670 | 1–9 | 1415 | 6 | 0 | 0 | 1409 | 46 |
|  |  |  | ≥15 | 1602 | 19 | 0 | 0 | 1583 | 39 |
|  |  |  | Total | 3547 | 37 | 0 | 0 | 3510 | 42 |
| Kunar | Asad Abad, Marawara | 10651 | 1–9 | 1555 | 3 | 0 | 0 | 1552 | 49 |
|  |  |  | ≥15 | 1243 | 50 | 1 | 1 | 1191 | 26 |
|  |  |  | Total | 3274 | 54 | 1 | 1 | 3218 | 40 |
|  | Chawkay, Khas Kunar, Sirkanay | 10671 | 1–9 | 1721 | 3 | 0 | 0 | 1718 | 47 |
|  |  |  | ≥15 | 1479 | 18 | 0 | 0 | 1461 | 38 |
|  |  |  | Total | 3719 | 25 | 0 | 0 | 3694 | 43 |
| Kunduz | Imam Sahib | 10652 | 1–9 | 1295 | 0 | 11 | 0 | 1284 | 47 |
|  |  |  | ≥15 | 960 | 11 | 0 | 0 | 949 | 13 |
|  |  |  | Total | 2639 | 11 | 11 | 0 | 2617 | 33 |
|  | Kunduz | 10653 | 1–9 | 1305 | 1 | 14 | 0 | 1290 | 47 |
|  |  |  | ≥15 | 1199 | 15 | 0 | 0 | 1184 | 28 |
|  |  |  | Total | 2974 | 17 | 14 | 0 | 2943 | 38 |
|  | Ali Abad, Chahar Dara | 10672 | 1–9 | 1228 | 12 | 0 | 0 | 1216 | 51 |
|  |  |  | ≥15 | 1531 | 0 | 0 | 0 | 1531 | 35 |
|  |  |  | Total | 3295 | 12 | 0 | 0 | 3283 | 43 |
|  | Khan Abad | 10673 | 1–9 | 1404 | 1 | 0 | 0 | 1403 | 44 |
|  |  |  | ≥15 | 1243 | 22 | 0 | 0 | 1221 | 33 |
|  |  |  | Total | 3061 | 25 | 0 | 0 | 3036 | 39 |
| Laghman | Mihtarlam | 10654 | 1–9 | 1228 | 1 | 8 | 0 | 1219 | 48 |
|  |  |  | ≥15 | 1071 | 22 | 0 | 0 | 1049 | 31 |
|  |  |  | Total | 2698 | 24 | 8 | 0 | 2666 | 40 |
|  | Qarghayi | 10655 | 1–9 | 1891 | 11 | 0 | 0 | 1880 | 46 |
|  |  |  | ≥15 | 1757 | 8 | 1 | 3 | 1745 | 44 |
|  |  |  | Total | 3651 | 19 | 1 | 3 | 3628 | 45 |
| Logar | Puli Alam | 10656 | 1–9 | 1773 | 11 | 0 | 0 | 1762 | 44 |
|  |  |  | ≥15 | 1222 | 26 | 7 | 6 | 1183 | 35 |
|  |  |  | Total | 3026 | 37 | 7 | 6 | 2976 | 40 |
|  | Khushi, Muhammad Agha | 10674 | 1–9 | 1351 | 3 | 0 | 0 | 1348 | 48 |
|  |  |  | ≥15 | 1367 | 0 | 0 | 0 | 1367 | 19 |
|  |  |  | Total | 3272 | 3 | 0 | 0 | 3269 | 33 |
| Nangarhar | Dara-I-Nur, Kama, Kuz Kunar | 10657 | 1–9 | 2012 | 33 | 5 | 4 | 1970 | 47 |
|  |  |  | ≥15 | 1513 | 20 | 252 | 194 | 1047 | 37 |
|  |  |  | Total | 3530 | 54 | 257 | 198 | 3021 | 44 |
| Nimroz | Chakhansur, Zaranj | 10658 | 1–9 | 1225 | 6 | 0 | 0 | 1219 | 50 |
|  |  |  | ≥15 | 1195 | 43 | 8 | 0 | 1144 | 54 |
|  |  |  | Total | 2821 | 59 | 8 | 0 | 2754 | 51 |
| Paktya | Ahmadaba, Jaji, Lija Ahmad Khel | 10659 | 1–9 | 1457 | 0 | 0 | 0 | 1457 | 46 |
|  |  |  | ≥15 | 701 | 26 | 2 | 0 | 673 | 25 |
|  |  |  | Total | 2560 | 26 | 2 | 0 | 2532 | 38 |
|  | Gardez, Jadran, Shwak | 10660 | 1–9 | 1385 | 1 | 0 | 0 | 1384 | 42 |
|  |  |  | ≥15 | 795 | 45 | 2 | 0 | 748 | 19 |
|  |  |  | Total | 2638 | 48 | 2 | 0 | 2588 | 35 |
| Samangan | Aybak, Dara-I-Sufi Payan, Feroznakhchir | 10661 | 1–9 | 984 | 1 | 18 | 0 | 965 | 50 |
|  |  |  | ≥15 | 1385 | 37 | 7 | 0 | 1341 | 47 |
|  |  |  | Total | 2705 | 38 | 25 | 0 | 2642 | 48 |
|  | Dara-I-Sufi Balla, Khuram Wa Sarbagh, Ruyi Du Ab | 10675 | 1–9 | 983 | 0 | 29 | 0 | 954 | 48 |
|  |  |  | ≥15 | 1861 | 28 | 6 | 0 | 1827 | 47 |
|  |  |  | Total | 3300 | 29 | 37 | 0 | 3234 | 48 |
| Sari Pul | Sangcharak, Sozma Qala | 10662 | 1–9 | 1324 | 4 | 0 | 0 | 1320 | 49 |
|  |  |  | ≥15 | 1058 | 0 | 0 | 0 | 1058 | 45 |
|  |  |  | Total | 2668 | 6 | 0 | 0 | 2662 | 48 |
|  | Sari Pul, Sayyad | 10663 | 1–9 | 1358 | 5 | 0 | 0 | 1353 | 50 |
|  |  |  | ≥15 | 930 | 4 | 0 | 0 | 926 | 34 |
|  |  |  | Total | 2472 | 9 | 0 | 0 | 2463 | 43 |
| Takhar | Taluqan | 10664 | 1–9 | 971 | 0 | 8 | 0 | 963 | 48 |
|  |  |  | ≥15 | 1486 | 119 | 78 | 3 | 1286 | 53 |
|  |  |  | Total | 2847 | 125 | 89 | 3 | 2630 | 51 |
|  | Bangi, Chal | 10676 | 1–9 | 900 | 0 | 8 | 0 | 892 | 47 |
|  |  |  | ≥15 | 1766 | 22 | 10 | 0 | 1734 | 46 |
|  |  |  | Total | 3068 | 25 | 20 | 0 | 3023 | 47 |
|  | Chah Ab, Yangi Qala | 10677 | 1–9 | 1011 | 1 | 12 | 0 | 998 | 51 |
|  |  |  | ≥15 | 1772 | 12 | 5 | 0 | 1755 | 46 |
|  |  |  | Total | 3308 | 16 | 18 | 0 | 3274 | 49 |
|  | Farkhar, Kalafgan | 10678 | 1–9 | 1009 | 0 | 21 | 0 | 988 | 50 |
|  |  |  | ≥15 | 1817 | 26 | 8 | 0 | 1783 | 48 |
|  |  |  | Total | 3375 | 28 | 30 | 0 | 3317 | 48 |

**Supplementary Table 2**. Household-level coverage of water, sanitation and hygiene facilities in 35 evaluation units (EUs) in Afghanistan, September 2018–December 2019.

| **District(s) in EU** | **Number of clusters visited** | **Total number of households surveyed** | **Households with improved drinking water source (%)** | **Households with improved washing water source (%)** | **Households with drinking water source within a 30-minute return journey of the house (%)** | **Households with washing water source within a 30-minute return journey of the house (%)** | **Households with an improved latrine (%)** | **Households with a latrine with a handwash station (%)** |
| --- | --- | --- | --- | --- | --- | --- | --- | --- |
| Baghlani Jadid | 22 | 547 | 366 (67) | 186 (34) | 443 (81) | 442 (81) | 67 (12) | 286 (52) |
| Nahrin, Puli Khumri | 22 | 482 | 206 (43) | 151 (31) | 456 (95) | 467 (97) | 164 (34) | 26 (5) |
| Bamyan, Kahmard, Sayghan, Shibar | 22 | 510 | 297 (58) | 264 (52) | 487 (95) | 490 (96) | 185 (36) | 193 (38) |
| Gizab, Kijran, Kiti, Nili | 22 | 507 | 263 (52) | 259 (51) | 480 (95) | 481 (95) | 104 (21) | 203 (40) |
| Bilchiragh, Maymana | 22 | 545 | 412 (76) | 391 (72) | 461 (85) | 461 (85) | 42 (8) | 37 (7) |
| Ghoryan, Kohsan | 22 | 552 | 396 (72) | 384 (70) | 491 (89) | 492 (89) | 108 (20) | 100 (18) |
| Hesa Awal Kohistan, Hesa Duwum Kohistan | 22 | 464 | 196 (42) | 102 (22) | 462 (100) | 463 (100) | 266 (57) | 49 (11) |
| Asad Abad, Marawara | 22 | 560 | 462 (83) | 355 (63) | 527 (94) | 525 (94) | 211 (38) | 38 (7) |
| Imam Sahib | 22 | 516 | 314 (61) | 242 (47) | 488 (95) | 498 (97) | 15 (3) | 44 (9) |
| Kunduz | 22 | 521 | 145 (28) | 120 (23) | 461 (88) | 469 (90) | 24 (5) | 16 (3) |
| Mihtarlam | 22 | 549 | 279 (51) | 264 (48) | 466 (85) | 467 (85) | 57 (10) | 34 (6) |
| Qarghayi | 22 | 555 | 195 (35) | 49 (9) | 449 (81) | 458 (83) | 155 (28) | 5 (1) |
| Puli Alam | 22 | 529 | 474 (90) | 426 (81) | 510 (96) | 511 (97) | 27 (5) | 14 (3) |
| Dara-I-Nur, Kama, Kuz Kunar | 22 | 559 | 518 (93) | 432 (77) | 527 (94) | 552 (99) | 157 (28) | 104 (19) |
| Chakhansur, Zaranj | 22 | 549 | 219 (40) | 200 (36) | 528 (96) | 532 (97) | 241 (44) | 250 (46) |
| Ahmadaba, Jaji, Lija Ahmad Khel | 22 | 547 | 456 (83) | 385 (70) | 517 (95) | 543 (99) | 14 (3) | 29 (5) |
| Gardez, Jadran, Shwak | 22 | 544 | 495 (91) | 409 (75) | 544 (100) | 544 (100) | 29 (5) | 9 (2) |
| Aybak, Dara-I-Sufi Payan, Feroznakhchir | 22 | 554 | 246 (44) | 244 (44) | 319 (58) | 329 (59) | 42 (8) | 47 (8) |
| Sangcharak, Sozma Qala | 22 | 529 | 274 (52) | 274 (52) | 186 (35) | 187 (35) | 71 (13) | 510 (96) |
| Sari Pul, Sayyad | 22 | 494 | 269 (54) | 272 (55) | 201 (41) | 200 (40) | 43 (9) | 337 (68) |
| Taluqan | 22 | 558 | 439 (79) | 409 (73) | 457 (82) | 481 (86) | 187 (34) | 187 (34) |
| Dushi, Khinjan | 22 | 548 | 523 (95) | 58 (11) | 547 (100) | 548 (100) | 58 (11) | 0 (0) |
| Panjab, Yakawlang | 22 | 549 | 316 (58) | 314 (57) | 487 (89) | 487 (89) | 174 (32) | 508 (93) |
| Miramor, Shahristan | 22 | 549 | 549 (100) | 515 (94) | 546 (99) | 546 (99) | 94 (17) | 8 (1) |
| Andkhoy, Qorghan | 22 | 550 | 316 (57) | 484 (88) | 544 (99) | 544 (99) | 50 (9) | 15 (3) |
| Guzara, Zinda Jan | 22 | 550 | 395 (72) | 394 (72) | 507 (92) | 508 (92) | 251 (46) | 507 (92) |
| Alasay, Nijrab | 22 | 551 | 466 (85) | 192 (35) | 551 (100) | 551 (100) | 48 (9) | 0 (0) |
| Chawkay, Khas Kunar, Sirkanay | 22 | 550 | 539 (98) | 428 (78) | 534 (97) | 536 (97) | 31 (6) | 33 (6) |
| Ali Abad, Chahar Dara | 22 | 548 | 388 (71) | 388 (71) | 548 (100) | 548 (100) | 109 (20) | 541 (99) |
| Khan Abad | 22 | 550 | 276 (50) | 54 (10) | 528 (96) | 528 (96) | 34 (6) | 0 (0) |
| Khushi, Muhammad Agha | 22 | 549 | 498 (91) | 496 (90) | 549 (100) | 549 (100) | 126 (23) | 506 (92) |
| Dara-I-Sufi Balla, Khuram Wa Sarbagh, Ruyi Du Ab | 22 | 550 | 243 (44) | 253 (46) | 515 (94) | 521 (95) | 39 (7) | 21 (4) |
| Bangi, Chal | 22 | 550 | 240 (44) | 196 (36) | 467 (85) | 470 (85) | 44 (8) | 27 (5) |
| Chah Ab, Yangi Qala | 22 | 549 | 342 (62) | 332 (60) | 428 (78) | 443 (81) | 20 (4) | 17 (3) |
| Farkhar, Kalafgan | 22 | 550 | 464 (84) | 430 (78) | 515 (94) | 515 (94) | 42 (8) | 28 (5) |
| *Total* | *770* | *18,864* | 12,476 (66) | 10,352 (55) | 16,726 (89) | 16,886 (90) | 3,329 (18) | 4,729 (25) |
| EU: Evaluation unit | | | | | | | | |
